# Supplementary material for: Geographic Inequalities of Respiratory Health Services Utilization during Childhood in Edmonton and Calgary, Canada: A Tale of Two Cities
Source: Int J Environ Res Public Health. 2020 Dec 2;17(23):8973. doi: 10.3390/ijerph17238973 (PMC7730300; doi:10.3390/ijerph17238973)
Supplement: Supplementary file 1 [file ijerph-17-08973-s001.pdf]

Supplementary Materials

Manuscript: Geographic inequalities of respiratory health services utilization during childhood in Edmonton and Calgary, Canada: a tale of two cities

Jesus Serrano-Lomelin, Charlene Nielsen, Anne Hicks, Susan Crawford, Jeffrey Bakal, and Maria B. Ospina.

Section S1. Standardized Prevalence Ratios (SPR) & smoothed SPR

Respiratory events = number of respiratory health service utilization (hospitalizations + ED visits) associated with a primary diagnosis of acute bronchiolitis, asthma, croup, influenza, pneumonia, other acute lower respiratory tract infections, and/or other acute upper respiratory tract infections.

SPR = Standardized Prevalence Ratio applying the indirect standardization method

Smoothed SPR = smoothed SPR

Y_{DA} = respiratory events by DA

Overall rate of Y for Alberta = 1.36

Expected Y_{DA} = number of live births * 1.36

SPR = Y_{DA} / *Expected* Y_{DA}

The smoothed SPR were obtained from empirical Bayes predictions using the Stata programs (commands) developed by Rabe-Hesketh and Skronda, 2008. Details in: Rabe-Hesketh, S., Skrondal, A., 2008. Multilevel and longitudinal modeling using Stata. 2nd ed. Stata Press, Texas.

Commands:
*** random-intercept (at DA level) Poisson regression ***
gllamm Sum_TotEvents, i(DAUID_2006) offset (lne) family (poisson) link(log) adapt
*** empirical Bayes predictions using gllapred command with “mu” option to get posterior means ***
gllapred mu, mu nooffset

Distribution of SPR and smoothed SPR by DA for Alberta

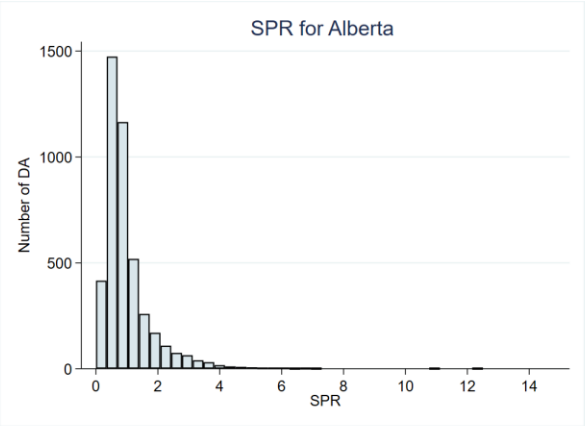

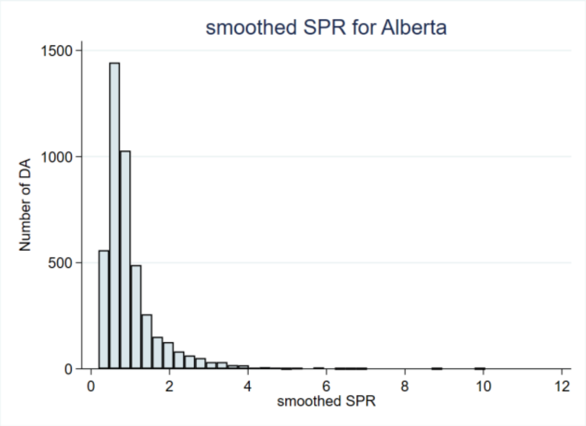

Section S2. Moran Scatter Plots.

Software: ESF-Tools. Technical details in: Griffith DA, Chun Y, Li B. Spatial Regression Analysis Using Eigenvector Spatial Filtering. Elservier Academic Press. 2019. E-book. Available online: https://doi.org/10.1016/B978-0-12-815043-6.09990-0

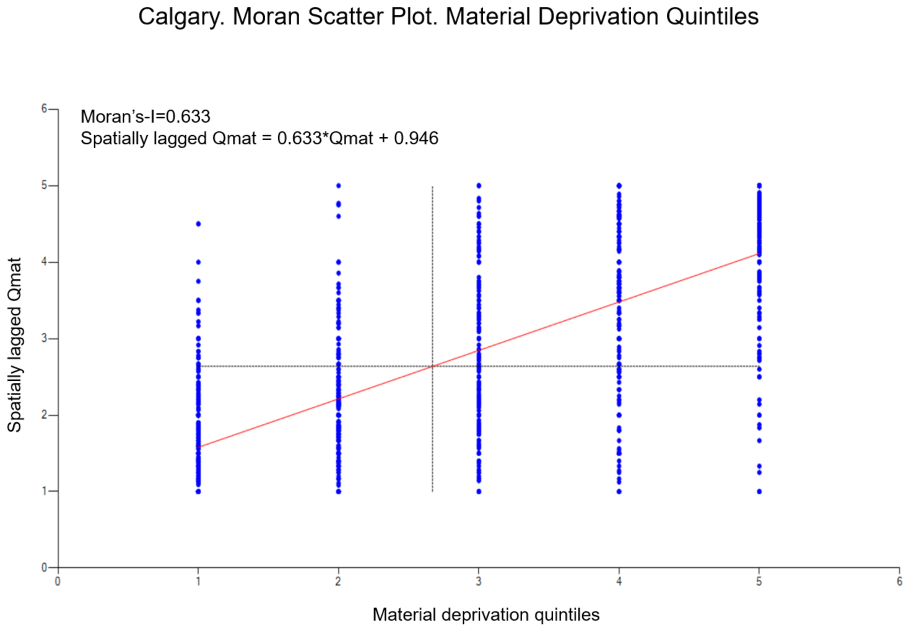

Figure S1. Moran scatter plot for the material deprivation quintiles, Calgary.

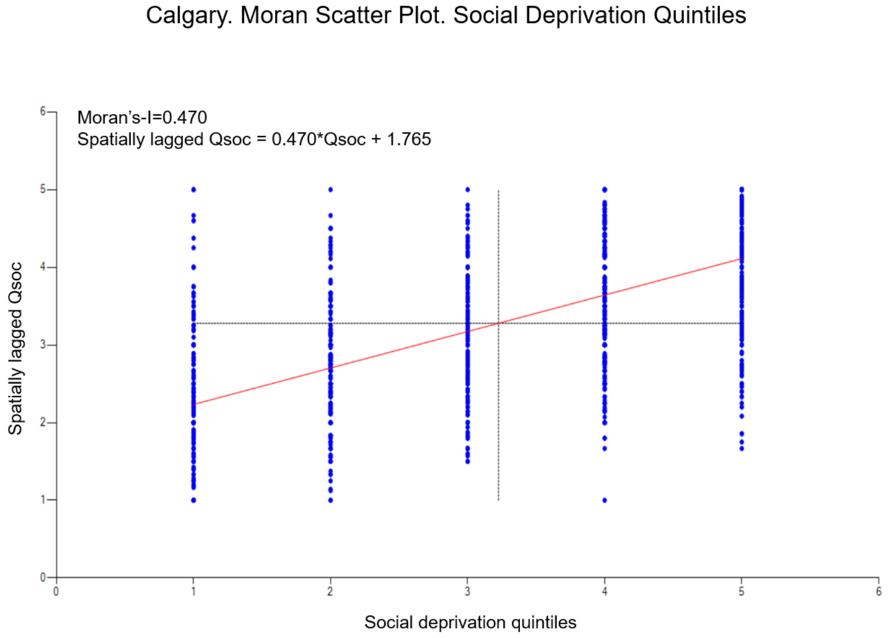

Figure S2. Moran scatter plot for the social deprivation quintiles, Calgary.

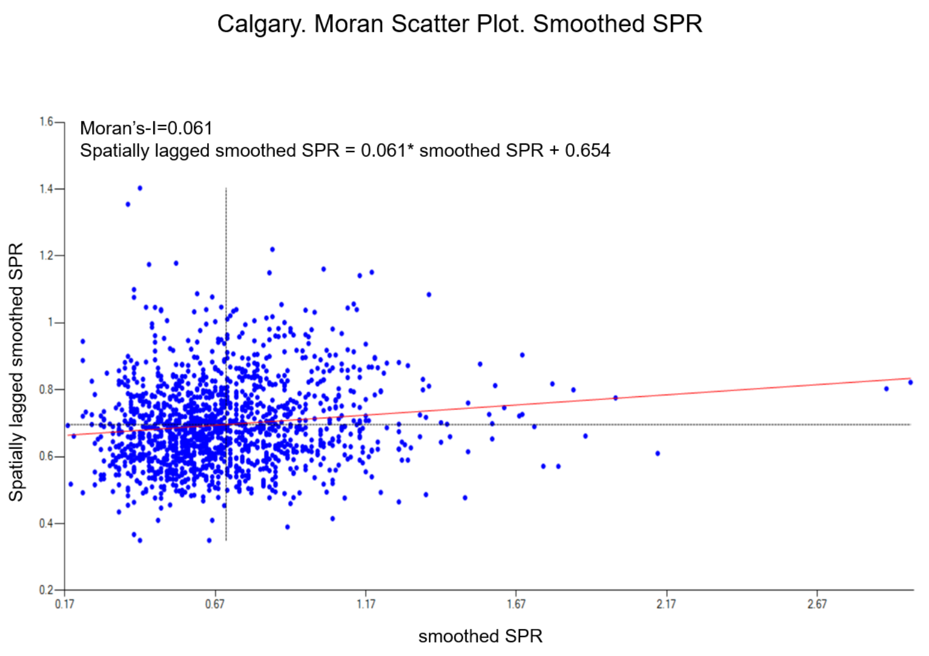

Figure S3. Moran scatter plot for the smoothed standardized prevalence ratios (SPR), Calgary.

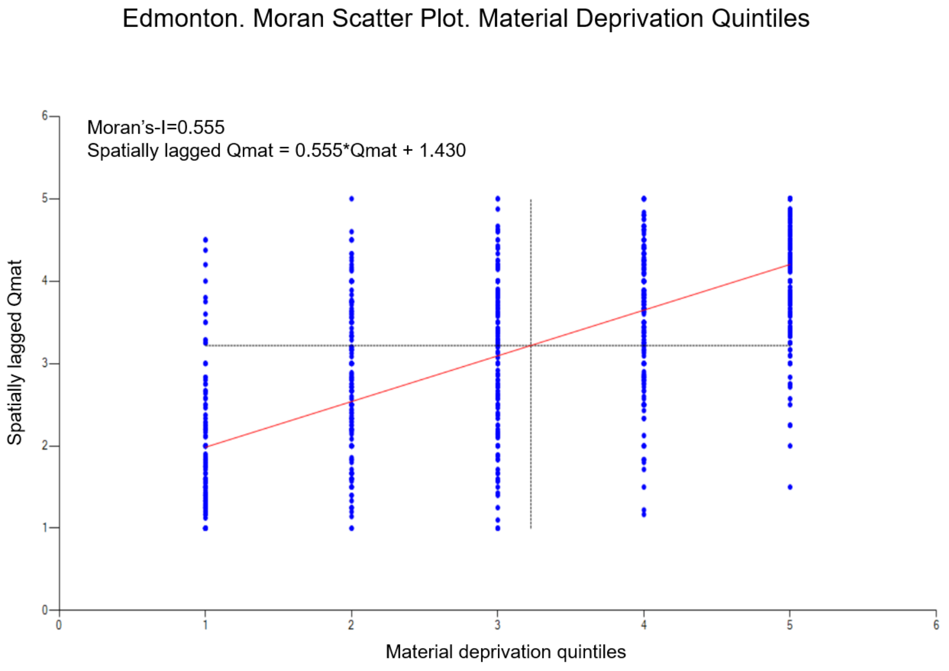

Figure S4. Moran scatter plot for the material deprivation quintiles, Edmonton.

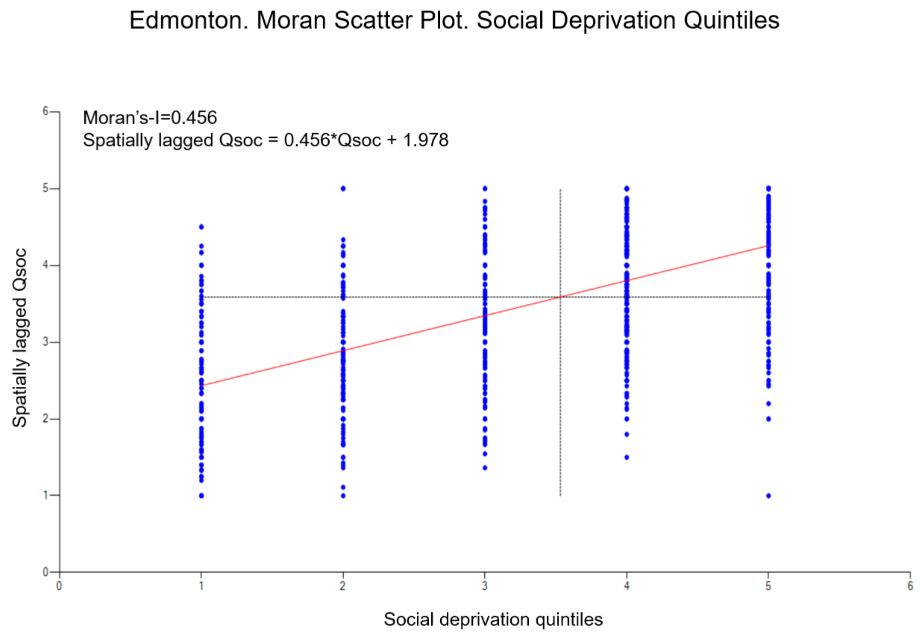

Figure S5. Moran scatter plot for the social deprivation quintiles, Edmonton.

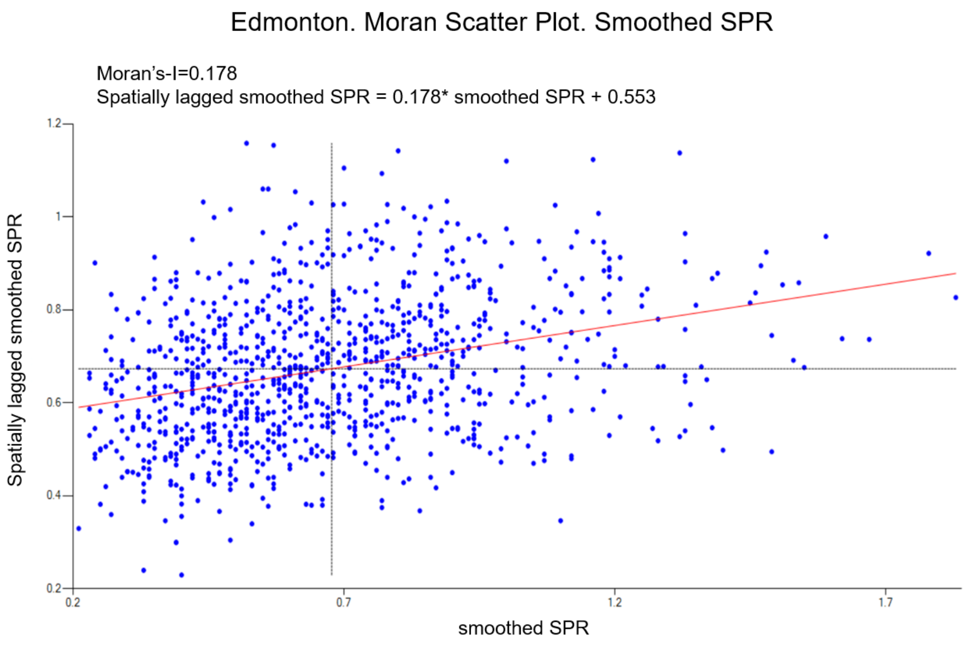

Figure S6. Moran scatter plot for the smoothed standardized prevalence ratios (SPR), Edmonton.

Section S3. Eigenvectors related to smoothed SPR

Calgary:

Initial solution:
Extraction of Eigenvectors (EVs) related to Y (smoothed SPR).
Linear regression model (family: gaussian)
Number of rows: 1403 (= number of DAs)
Number of candidate EVs: **263.**
MC (Morans'-I coefficient) of non-ESF residuals: 0.058, p-value < 0.001
AIC of non-ESF: -3,639.44, AIC of Final Model: -3,782.06
Residual standard error: 0.25 on 1348 degrees of freedom
Multiple R-squared: 0.17, Adjusted R-squared: 0.14
F-Statistic: 5.21 on 54 and 1348 DF, p-value < 0.001
MC (Morans'-I coefficient) of residuals: -0.112, p-value > 0.999

Cumulative coefficient of determination (R-squared) for the first 20 eigenvectors and eigenvectors selected by changes in the increment rate of R-squared.

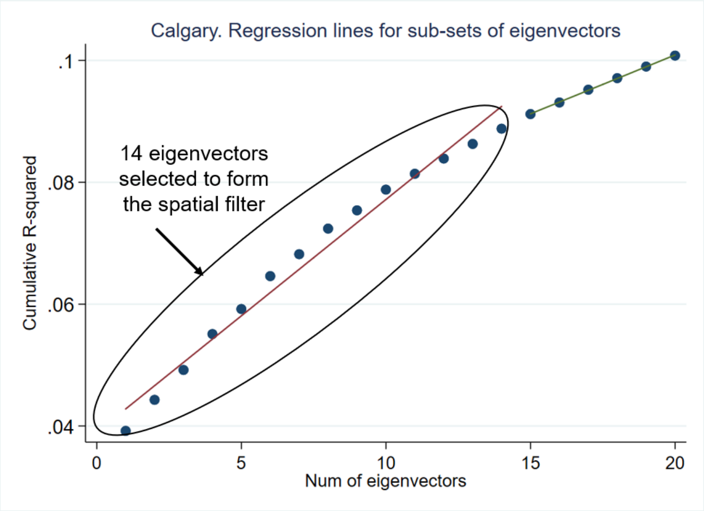

Figure S7. Cumulative R^2 for the first 20 selected eigenvectors, Calgary.

Regression model for the first 20 eigenvectors (Calgary):

```
. regress bayesSIR i.qmat i.qsoc PM25 NO2 EV20 EV100 EV37 EV254 EV2 EV19 EV3 EV90 EV30 EV5 EV4 EV223
>  EV34 EV89 EV220 EV13 EV101 EV165 EV29 EV15

      Source |       SS           df       MS      Number of obs   =     1,403
-------------+----------------------------------   F(30, 1372)     =      6.24
       Model |  12.7008123        30  .423360411   Prob > F        =    0.0000
    Residual |  93.1452423     1,372  .067890118   R-squared       =    0.1200
-------------+----------------------------------   Adj R-squared   =    0.1008
       Total |  105.846055     1,402  .075496473   Root MSE        =    .26056

------------------------------------------------------------------------------
    bayesSIR |      Coef.   Std. Err.      t    P>|t|     [95% Conf. Interval]
-------------+----------------------------------------------------------------
        qmat |
          2  |   .0328599   .0210212     1.56   0.118    -.0083772     .074097
          3  |   .0328482   .0219468     1.50   0.135    -.0102048    .0759011
          4  |  -.0062995   .0235242    -0.27   0.789    -.0524469    .0398478
          5  |    .052593   .0217882     2.41   0.016     .0098513    .0953347
             |
        qsoc |
          2  |   .0206011   .0245187     0.84   0.401    -.0274971    .0686993
          3  |   .0219702    .023562     0.93   0.351    -.0242513    .0681916
          4  |   .0291058   .0232329     1.25   0.210      -.01647    .0746815
          5  |   .0819495   .0235883     3.47   0.001     .0356765    .1282226
             |
        PM25 |   .0225614   .0313875     0.72   0.472    -.0390112    .0841341
         NO2 |  -.0010719   .0026306    -0.41   0.684    -.0062324    .0040886
        EV20 |   1.223901   .2646936     4.62   0.000     .7046534     1.74315
       EV100 |  -.8584619   .2634663    -3.26   0.001    -1.375302   -.3416216
        EV37 |   .8222507   .2645451     3.11   0.002      .303294    1.341207
       EV254 |  -.8402148    .261576    -3.21   0.001    -1.353347   -.3270825
         EV2 |  -.9035374   .2933771    -3.08   0.002    -1.479054   -.3280212
        EV19 |  -.8027666   .2642613    -3.04   0.002    -1.321167   -.2843666
         EV3 |   .7610725   .2741106     2.78   0.006     .2233513    1.298794
        EV90 |   .6974374   .2627518     2.65   0.008     .1819986    1.212876
        EV30 |   .6280187   .2659737     2.36   0.018     .1062594    1.149778
         EV5 |   .6201274   .2665339     2.33   0.020     .0972692    1.142985
         EV4 |   .5977357   .2644727     2.26   0.024     .0789211     1.11655
       EV223 |  -.5885674   .2612139    -2.25   0.024    -1.100989   -.0761454
        EV34 |  -.5799219   .2623382    -2.21   0.027    -1.094549   -.0652945
        EV89 |   .5755243    .263821     2.18   0.029     .0579881     1.09306
       EV220 |   .5644067   .2617677     2.16   0.031     .0508984    1.077915
        EV13 |   .5586133   .2796462     2.00   0.046      .010033    1.107194
       EV101 |  -.5350212   .2613683    -2.05   0.041    -1.047746   -.0222964
       EV165 |  -.5233198   .2613052    -2.00   0.045    -1.035921   -.0107188
        EV29 |  -.5153174   .2622658    -1.96   0.050    -1.029803    -.000832
        EV15 |  -.5054817   .2622411    -1.93   0.054    -1.019919    .0089552
       _cons |   .5153686   .2035172     2.53   0.011       .11613    .9146072
------------------------------------------------------------------------------
```

Edmonton:

Initial solution:

Extraction of Eigenvectors (EVs) related to Y (smoothed SPR).
Linear regression model (family: gaussian)
Number of rows: 1038 (= number of DAs)
Number of candidate EVs: **214.**
MC (Morans'-I coefficient) of non-ESF residuals: 0.046, p-value = 0.005
AIC of non-ESF: -2,874.28, AIC of Final Model: -2,971.69
Residual standard error: 0.23 on 983 degrees of freedom
Multiple R-squared: 0.29, Adjusted R-squared: 0.25
F-Statistic: 7.35 on 54 and 983 DF, p-value < 0.001
MC (Morans'-I coefficient) of residuals: -0.108, p-value > 0.999

Cumulative coefficient of determination (R-squared) for the first 20 eigenvectors and eigenvectors selected by changes in the increment rate of R-squared.

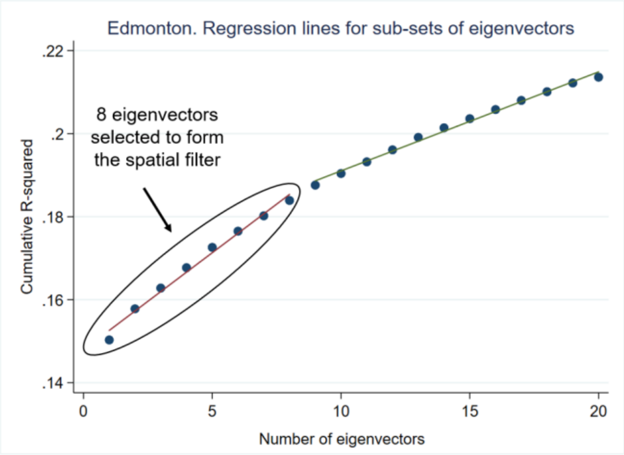

Figure S8. Cumulative R^2 for the first 20 selected eigenvectors, Edmonton.

Regression model for the first 20 eigenvectors (Edmonton):

```
. regress bayesSIR i.qmat i.qsoc PM25 NO2 EV2 EV17 EV9 EV55 EV50 EV22 EV15 EV184 EV67 EV194 EV139 EV
> 8 EV23 EV48 EV76 EV119 EV137 EV40  EV167 EV125
```

Source	SS	df	MS
Model	17.6937116	30	.589790387
Residual	57.1514652	1,006	.056810602
Total	74.8451769	1,036	.072244379

Number of obs	=	1,037
F(30, 1006)	=	10.38
Prob > F	=	0.0000
R-squared	=	0.2364
Adj R-squared	=	0.2136
Root MSE	=	.23835

bayesSIR	Coef.	Std. Err.	t	P>|t|	[95% Conf.	Interval]
qmat						
2	.0883148	.0263044	3.36	0.001	.036697	.1399327
3	.0863443	.0256084	3.37	0.001	.0360924	.1365962
4	.1701338	.0249313	6.82	0.000	.1212105	.219057
5	.186142	.0240064	7.75	0.000	.1390337	.2332503
qsoc						
2	.0543208	.0292169	1.86	0.063	-.0030122	.1116538
3	.0431703	.029035	1.49	0.137	-.0138057	.1001464
4	.0980844	.0271	3.62	0.000	.0449054	.1512635
5	.1642038	.0260537	6.30	0.000	.1130779	.2153297
PM25	-.0125378	.0111863	-1.12	0.263	-.0344889	.0094133
NO2	-.0014874	.0027885	-0.53	0.594	-.0069594	.0039845
EV2	-1.060505	.2614888	-4.06	0.000	-1.573631	-.5473794
EV17	-.79611	.2575656	-3.09	0.002	-1.301538	-.2906825
EV9	.8186166	.2645433	3.09	0.002	.2994967	1.337737
EV55	-.6688533	.240497	-2.78	0.006	-1.140787	-.1969201
EV50	.6575233	.2396957	2.74	0.006	.1871624	1.127884
EV22	.6117342	.2419903	2.53	0.012	.1368706	1.086598
EV15	.6091795	.2509173	2.43	0.015	.1167981	1.101561
EV184	-.5745379	.240562	-2.39	0.017	-1.046599	-.1024772
EV67	-.5728051	.2404366	-2.38	0.017	-1.04462	-.1009904
EV194	.5176566	.2392186	2.16	0.031	.048232	.9870813
EV139	-.5151429	.2396536	-2.15	0.032	-.9854211	-.0448646
EV8	-.5207671	.2436089	-2.14	0.033	-.9988069	-.0427274
EV23	.5061818	.242903	2.08	0.037	.0295271	.9828365
EV48	-.4874108	.2405062	-2.03	0.043	-.9593621	-.0154595
EV76	-.4774047	.2413736	-1.98	0.048	-.9510582	-.0037513
EV119	-.4723218	.2391255	-1.98	0.049	-.9415637	-.00308
EV137	-.4655904	.2395517	-1.94	0.052	-.9356687	.0044878
EV40	-.4738924	.245184	-1.93	0.054	-.9550231	.0072383
EV167	-.4574489	.2395661	-1.91	0.056	-.9275555	.0126577
EV125	-.405023	.2392616	-1.69	0.091	-.8745319	.064486
_cons	.5874285	.0954088	6.16	0.000	.4002055	.7746516

Section S4. Sensitivity analysis: comparison of predicted rates using queen vs. rook connectivity polygons.

The eigenvector spatial filtering (ESF) solution depends on technical specifications at several steps. In our analysis, the connectivity matrix of dissemination areas (DA) was defined from the "queen" rule, in which DA consider neighbours if they share boundaries based on a single point (node) or a segment of border limits. Another alternative is to use the "rook" rule. In the rook connectivity, polygons are neighbours is they share a segment of border.

We estimated the predicted standardized prevalence ratios (SPR), previously smoothed by empirical Bayes estimators, for both queen and rook definitions of the connectivity matrix. Candidate eigenvectors were chosen based on positive spatial correlation considering a minimum threshold of 0.25 of the Moran's index coefficient. The subset of eigenvectors selected to build the spatial filter, was based on maximization of R^2 in the regression model. The spatial filter values were divided into quintiles. Each quintile defined a geographic zone from which we estimated the average of the smoothed SPR (and 95% CI) to quantify geographic inequalities. The results for the spatial filters according to queen and rook connectivity matrices are presented in **Table S1** for Calgary, and **Table S2** for Edmonton. No substantial differences were observed between both approaches.

Table S1. Predicted standardized prevalence ratios (SPR) for both queen and rook definitions of the connectivity matrix in Calgary.

	Queen connectivity		Rook connectivity	
**Spatial Filter (quintiles)**	**Predicted SPR**	**95% CI**	**Predicted SPR**	**95% CI**
1	0.58	(0.55, 0.61)	0.59	(0.55, 0.62)
2	0.65	(0.62, 0.68)	0.62	(0.59, 0.65)
3	0.69	(0.66, 0.72)	0.70	(0.67, 0.73)
4	0.75	(0.72, 0.78)	0.76	(0.73, 0.79)
5	0.87	(0.83, 0.90)	0.87	(0.84, 0.90)

CI = confidence interval; SPR = standardized prevalence ratios

Table S2. Predicted standardized prevalence ratios (SPR) for both queen and rook definitions of the connectivity matrix in Edmonton.

	Queen connectivity		Rook connectivity	
**Spatial Filter (quintiles)**	**Predicted SPR**	**95% CI**	**Predicted SPR**	**95% CI**
1	0.57	(0.54, 0.61)	0.54	(0.50, 0.57)
2	0.60	(0.57, 0.64)	0.61	(0.58, 0.64)
3	0.68	(0.64, 0.71)	0.65	(0.62, 0.68)
4	0.74	(0.71, 0.78)	0.72	(0.69, 0.75)
5	0.79	(0.76, 0.82)	0.87	(0.84, 0.90)

CI = confidence interval; SPR = standardized prevalence ratios

Section S5. Maps of air pollutant concentrations. Nitrogen Dioxide (NO_2) and fine Particulate Matter ($PM_{2.5}$).

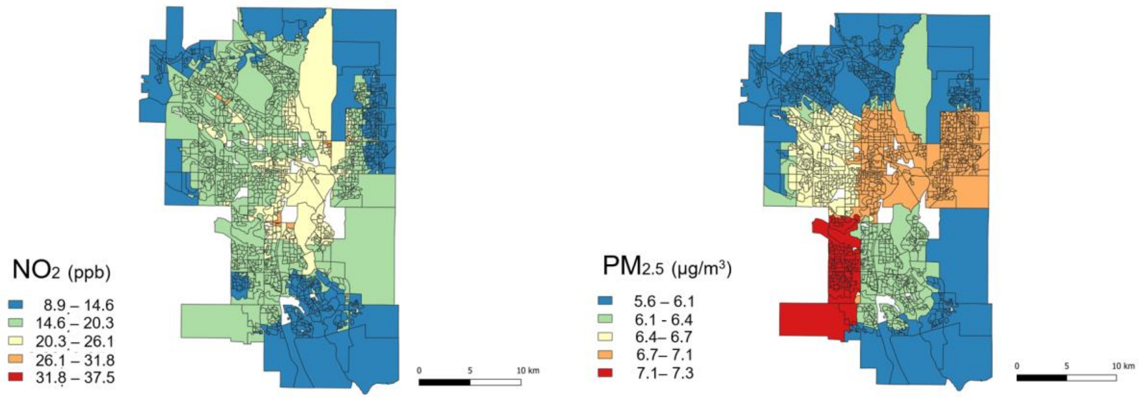

Figure S9. Air pollutant concentrations for NO_2 and $PM_{2.5}$ in Calgary.

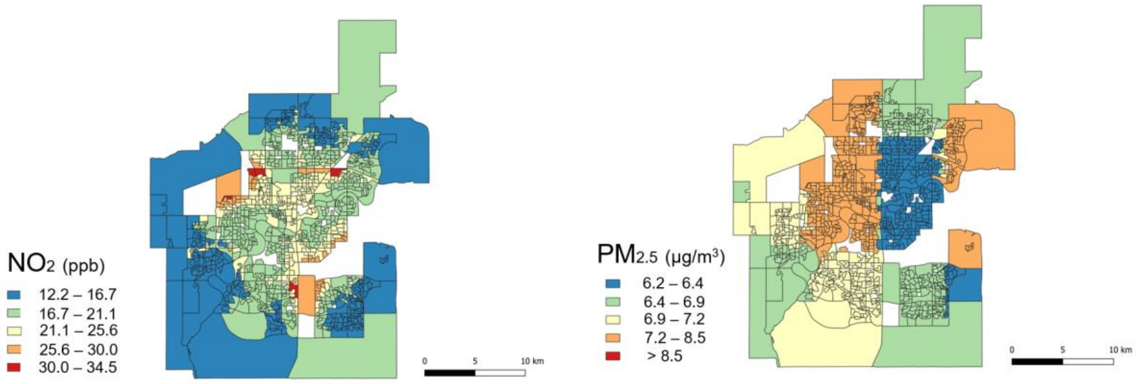

Figure S10. Air pollutant concentrations for NO_2 and $PM_{2.5}$ in Edmonton.

Air pollutant concentrations were reported by Hystad P, Setton E, Cervantes A, Poplawski K, Deschenes S, Brauer M, et al. Creating national air pollution models for population exposure assessment in Canada. *Environ. Health Perspect*. 2011;119:1123–1129. https://doi.org/10.1289/ehp.1002976
